# Postoperative Analgesic and Anti-inflammatory Effectiveness of Ginger (Zingiber officinale) and NSAIDs as Adjuncts to Nonsurgical Periodontal Therapy for the Management of Periodontitis

**DOI:** 10.3290/j.ohpd.b3125633

**Published:** 2022-06-13

**Authors:** Nouf Alshibani, Reem Al-Kattan, Lamees Alssum, Amani Basudan, Marwa Shaheen, Montaser N. Alqutub, Fahda Al Dahash

**Affiliations:** a Associate Professor, Department of Periodontics and Community Dentistry, College of Dentistry, King Saud University, Riyadh, Saudi Arabia. Designed and supervised the study, wrote the manuscript, revised and approved it prior to submission.; b Assistant Professor, Department of Periodontics and Community Dentistry, College of Dentistry, King Saud University, Riyadh, Saudi Arabia. Performed the clinical examinations, wrote the manuscript, revised and approved it prior to submission.; c Assistant Professor, Department of Periodontics and Community Dentistry, College of Dentistry, King Saud University, Riyadh, Saudi Arabia. Administered the questionnaire to the patients, wrote the manuscript, revised and approved it prior to submission.; d Assistant Professor, Department of Periodontics and Community Dentistry, College of Dentistry, King Saud University, Riyadh, Saudi Arabia. Performed the statistical analysis, wrote the manuscript, revised and approved it prior to submission.; e Assistant Professor, Department of Periodontics and Community Dentistry, College of Dentistry, King Saud University, Riyadh, Saudi Arabia. Wrote the manuscript, revised and approved it prior to submission.; f Associate Professor, Department of Periodontics and Community Dentistry, College of Dentistry, King Saud University, Riyadh, Saudi Arabia. Wrote the manuscript, revised and approved it prior to submission.; g Lecturer, Department of Oral and Maxillofacial Surgery and diagnostic Sciences, Riyadh Elm University, Riyadh, Saudi Arabia. Wrote the manuscript, revised and approved it prior to submission.

**Keywords:** inflammation, interleukin 1-beta, periodontal disease, probing depth, soluble urokinase plasminogen activator receptor

## Abstract

**Purpose:**

The authors hypothesize that ginger (*Zingiber officinale*) tablets and non-steroidal anti-inflammatory drugs (NSAIDs) are effective in reducing postoperative self-rated pain and periodontal parameters (plaque index [PI], gingival index [GI], and probing depth [PD], clinical attachment loss [AL] and marginal bone loss) following non-surgical periodontal therapy (NSPT) in patients with periodontitis. The aim was to compare the postoperative analgesic and anti-inflammatory effectiveness of ginger tablets and NSAIDs as adjuncts to nonsurgical periodontal therapy for the management of periodontitis.

**Materials and Methods:**

Patients with periodontitis were included. All patients underwent NSPT. In groups 1 and 2, patients received postoperative ginger (400 mg) and non-steroidal anti-inflammatory drugs (400 mg), respectively. Demographic data were collected, and full-mouth periodontal parameters (PI, GI, PD and CAL) were evaluated at baseline and at 7, 14 and 21 days. Self-rated pain scores were assessed at baseline, and at 24 h, 3 and 7 days of follow-up. In both groups, self-rated pain was assessed pre- and postoperatively using the numeric rating scale (NRS). Power analysis was performed on data from a pilot investigation and group comparisons were done. Statistical significance was set at p < 0.01.

**Results:**

Baseline mean NRS scores in groups 1 and 2 were 4.19 ± 0.12 and 4.13 ± 0.08, respectively. All participants had stage II/grade B periodontitis. At baseline, self-rated pain scores were significantly higher among patients in groups 1 and 2 at 24 h (p < 0.01) and 3 days (p < 0.01) of follow-up. In groups 1 (p < 0.01) and 2 (p < 0.01), self-rated pain scores were significantly higher at 24 h compared with 3 days of follow-up. In both groups, there was a significant reduction in PI (p < 0.01), GI (p < 0.01) and PD (p < 0.01) at 7, 14 and 21 days of follow-up compared with baseline.

**Conclusion:**

Ginger and traditional NSAIDs are effective in reducing postoperative pain and inflammation following NSPT in patients with moderate periodontitis.

Accumulation of dental plaque in supra- and subgingival areas is a risk factor for periodontal inflammation.^25^ Generalised pain in gingival tissues as well as pain during mastication and gingival bleeding are common complaints reported by patients with periodontal inflammation.^29,47^ In undiagnosed and/or untreated cases, periodontal inflammation worsens and may ultimately lead to tooth loss.^12,26,42,43^ Traditionally, nonsurgical periodontal therapy (NSPT) or scaling and root planing is commonly performed to treat periodontal inflammation.^4,11^ Non-steroidal anti-inflammatory drugs (NSAIDs) are often prescribed to patients after NSPT.^15^ It has been reported that NSAIDs as adjuncts to NSPT contribute towards reducing probing depth (PD), bleeding on probing (BoP), plaque index (PI) and gingival index (GI).^15,21^ Moreover, NSAIDs have also been shown to reduce postoperative pain after periodontal therapy.^13^

Complementary alternate medications (CAM) are usually derived from medicinal plants and are perceived to have no undesirable side-effects compared with synthetic pharmacological drugs.^8,27^ Patients often use CAM for the relief of pain, including pain in the orofacial region.^8,30^ *Zingiber officinale* (ginger) is a commonly used spice in food items; however, it is also used medicinally in many countries, including Saudi Arabia, India, Pakistan, and Iran.^1,3,32,48^ Active ingredients in ginger, such as gingerol, paradol, beta-bisabolene and shogaol, have been reported to exhibit anti-inflammatory, analgesic, anti-obesity, anti-viral and anti-oxidant effects.^9,14,34,44^ According to Mazidi et al,^33^ ginger reduces the production and serum levels of inflammatory cytokines including interleukin (IL)-6, IL-1beta and tumor necrosis factor alpha. In a recent cross-over randomised controlled trial (RCT), Menon et al^34^ compared the analgesic efficacy of NSAIDs (Ibuprofen 400 mg) and ginger (400 mg capsules) following surgical periodontal debridement among patients with periodontitis. In that study, all patients, GI and postoperative pain scores (using the visual analogue scale [VAS]) were assessed at baseline and for up to 2 days postoperatively. The results showed no statistically significant difference in GI and self-rated pain scores in the study groups. The authors concluded that ginger and NSAIDs are comparable in terms of their analgesic and anti-inflammatory effects following open-flap surgical periodontal debridement.^34^ However, there are no RCTs that have compared the efficacy of NSAIDs and ginger in terms of reduction in PI, probing depth (PD), clinical attachment loss (CAL), and self-rated pain scores following NSPT in patients with periodontal disease. The authors hypothesise that ginger tablets and NSAIDs are effective in reducing postoperative self-rated pain and periodontal parameters (plaque index [PI], GI, PD and CAL) following nonsurgical periodontal therapy (NSPT) in patients with periodontitis. The aim of this study was to compare the postoperative analgesic and anti-inflammatory effectiveness of ginger tablets and NSAIDs as adjuncts to nonsurgical periodontal therapy for the management of periodontitis.

## Materials and Methods

### Ethical Considerations

Guidelines documented in the Helsinki 2013 Declaration on experiments involving humans were adopted for this study. Participating individuals were mandated to read and signed a consent form. Prior to signing the consent form, all participating patients were informed that they could withdraw from the study at any stage without any penalty, and were invited to ask questions. The ethics committee of the Riyadh Elm University, Riyadh, Saudi Arabia approved the study protocol (FRP/2021/388/603).

### Inclusion and Exclusion Criteria

Periodontal inflammation was defined as the presence of at least 6 periodontal pockets of at least 4 mm in each jaw quadrant with accompanying BOP.^39,40^ Individuals habitually using alcohol and nicotinic products such as smokeless tobacco, waterpipe, cigarettes, pipe, cigarillos and electronic nicotine delivery systems (ENDS) were excluded. Patients with self-reported systemic diseases, such as diabetes mellitus (DM), cardiomyopathy, obesity, HIV/AIDS, renal diseases and hepatic disorders, were excluded. Third molars, supernumerary teeth and grossly carious teeth were not assessed. Furthermore, pregnant/lactating individuals and individuals using bisphosphonates, probiotics, antibiotics, and steroids were not sought.

### Grouping

Therapeutically, patients were divided into 2 groups. In groups 1 and 2, patients received postoperative ginger (400 mg) (MEPACO Medifood, Product-code 11656; Beaver Dam, WI, USA) and Ibuprofen (400 mg) (Motrin IB, McNeil Consumer Healthcare; Fort Washington, PA, USA), respectively. In groups 1 and 2, patients were advised to take the respective tablets every 12 h for 3 days and then as needed for pain.

### Nonsurgical Periodontal Therapy

In all patients, NSPT was performed using an ultrasonic hand-scaler (Dental Equipment Woodpecker Uds-J Ultrasonic Scaler EMS Compatible Original; Guangzhou, China) and sterile curettes (Hu-Friedy; Chicago, IL, USA). All participants were instructed to postoperatively rinse with 15 ml of 0.12% chlorhexidine gluconate twice daily for 2 weeks.

### Questionnaire and Assessment of Self-rated Pain

Demographic data pertaining to age, gender, family history of gum disease, pre- and postoperative self-rated pain were collected using a questionnaire. Patients used a numeric rating scale ranging from 0 to 10 to self-assess their pre- and postoperative pain.^35^ Pain levels were allocated to one of 4 scores: 0 = no pain; 1-3 = mild pain; 4-6 = moderate pain; and 7-10 = severe pain.^35^ Self-rated pain scores were assessed at baseline, 24 h, 3 days and 1 week of follow-up.

### Periodontal Parameters

In test and control groups, full-mouth PI,^31^ GI,^31^ CAL,^6^ and PD^7^ and were measured by an investigator (Kappa 0.94). PD was measured to the nearest mm using a UNC-15 periodontal probe (Hu-Friedy). Marginal bone loss (MBL) on mesial and distal surfaces of all teeth was measured on digital intraoral radiographs by one calibrated investigator. The MBL was defined as the linear distance from 2 mm below the cementoenamel junction to the alveolar crest.^25^ The number of missing teeth was also recorded in all groups. Periodontal parameters were evaluated at baseline and at 1, 3 and 6 weeks of follow-up. Staging and grading of periodontitis was done as described elsewhere.^37^

### Allocation Concealment and Blinding

Opaque envelops were used to conceal group allocation. The principal investigator was responsible for group allocation and randomisation of patients. A calibrated and trained investigator blinded to the study groups administered the questionnaire to the participants and performed NSPT and periodontal evaluations.

### Follow-up

In all groups, the patients were evaluated for self-rated pain scores at 24 h and 3 and 7 days of follow-up. Periodontal parameters were assessed at baseline and at 7, 14 and 21 days of follow-up.

### Statistical and Power Analysis

Statistical software (SPSS, version 20; Chicago, IL, USA) was used to perform group comparisons in relation to clinical parameters and self-rated pain scores. The Kolmogrov-Smirnov test was used to assess data normality. Group-wise statistical evaluations were done using the non-parametric paired t-test and the Mann-Whitney U-test. Self-rated pain scores and periodontal parameters at baseline and at follow-up were compared using one-way ANOVA and Bonferroni post-hoc adjustment. Correlation between age, gender and periodontal parameters (PI, GI, CAL, PD and MBL) was assessed using logistic regression models. p < 0.01 was considered statistically significant. Prior sample-size estimation was done using data obtained from a pilot investigation (nQuery Advisor 5, Statistical Solutions; Saugus, MA, USA). Sample size estimation was based on the premise that a mean difference of 1 mm in CAL and PD would be detected at a significance level of 0.01, with a desired study power of at least 90%. It was estimated that a sample size of at least 20 patients/group would achieve 90% study power with a 0.01 two-sided significance level.

## Results

### Study Groups

Twenty-two (18 males and 4 females) and 22 (16 males and 6 females) individuals were included in groups 1 and 2, respectively. The mean age of individuals in groups 1 and 2 were 55.6 ± 3.1 and 53.7 ± 1.8 years, respectively. A family history of ‘gum disease’ was reported by 40.9% and 22.7% individuals in groups 1 and 2, respectively. Eight (36.4%) individuals in group 1 and 10 (45.5%) in group 2 were unaware if they had a family history of ‘gum disease’ (Table 1).

**Table 1:** Characteristics of the study groups

Parameters	Group 1	Group 2
Participants (n)	22	22
Male:Female	18:4	16:6
Mean age in years	45.6 ± 1.8	47.7 ± 0.4
Family history of gum disease		
Yes (n) (%)	9 (40.9%)	5 (22.7%)
No (n) (%)	5 (22.7%)	7 (31.8%)
Don’t know (n) (%)	8 (36.4%)	10 (45.5%)

### Self-rated Pain Scores at Baseline and at Follow-up

At baseline, self-rated mean pain scores in groups 1 and 2 were 4.19 ± 0.12 and 4.13 ± 0.08, respectively. At baseline, self-rated pain scores were statistically significantly higher among patients in groups 1 and 2 at 24 h (p < 0.01) and 3 days (p < 0.01) of follow-up. In groups 1 (p < 0.01) and 2 (p < 0.01), self-rated pain scores were statistically significantly higher at 24 h compared with 3 days of follow-up. None of the individuals in groups 1 and 2 reported to be in any pain and/or discomfort 7 days postoperatively (Table 2).

**Table 2:** Self-rated pain scores at baseline and at 24 h, and 3 and 7 days of follow-up

Study groups	Baseline	Follow-up		
		24 h	3 days	7 days
Group 1	4.19 ± 0.12*	1.3 ± 0.1†	0.2 ± 0.03	0
Group 2	4.13 ± 0.08*	1.5 ± 0.08†	0.3 ± 0.02	0

*Compared with 24 h and 3 days of follow-up (p < 0.01). †Compared with 3 days of follow-up (p < 0.01).

### Periodontal Parameters at Baseline and at Follow-up

**Fig 1:**
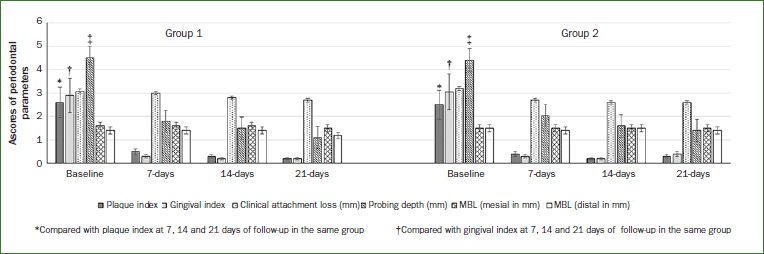
Periodontal parameters in groups 1 and 2 at baseline and at 7, 14 and 21 days of follow-up.

At baseline, participants in groups 1 and 2 had stage II/grade B periodontitis, with no statistically significant difference in PI, GI, CAL, PD, and mesial and distal MBL in groups 1 and 2 (Fig 1). In groups 1 and 2, there was a statistically significant reduction in PI (p < 0.01), GI (p < 0.01) and PD (p < 0.01) at 7, 14 and 21 days of follow-up compared with their respective baseline values. When compared with group 2, there was no statistically significant difference in reductions in scores of PI, GI, CAL, PD, mesial and distal MBL among patients in froup 1 (Fig 1). The numbers of missing teeth in groups 1 and 2 were 7.1 ± 0.5 and 6.5 ± 0.2, respectively. As per dental records, these teeth were extracted, as they had non-restorable dental carious lesions. None of the missing teeth were extracted for periodontal reasons.

### Correlation Between Pain and Periodontal Parameters

In group 1 and 2, there was no statistically significant correlation between self-rated pain and age, gender or periodontal parameters (PI, GI, CAL, PD and MBL) (data not shown).

## Discussion

The present results showed that ginger and NSAIDs are effective in reducing postoperative pain after NSPT, with no statistically significant difference in the analgesic efficacy between the two. These results support the study hypothesis, as a statistically significant reduction in PI, GI and PD was observed in both groups (Fig 1). Abundant evidence in indexed literature has shown that ginger is a potent CAM that can successfully be used for the management of inflammatory conditions such osteoarthritis, oral aphthous stomatitis and mucositis.^5,18-20^ Moreover, ginger has also been shown to reduce severe lower abdominal pain, a common manifestation during dysmenorrhea.^45^ The exact mechanism through which ginger exerst its analgesic effects remains unclear; however, results from preclinical studies have shown that ginger contains several compounds, such as capsacine, curcumin, gingerols, beta-carotene, and caffeic acid that exhibit antibacterial, antiviral, anti-pyretic, anti-inflammatory and analgesic properties.^5,17^ Moreover, ginger modulates pain by suppressing the production of prostaglandins via inhibition of lipoxygenase (LOX) and cyclooxygenase (COX) pathways.^41^ Here, it is pertinent to mention that NSAIDs also exert their anti-inflammatory and analgesic effects by inhibiting COX/LOX pathways.^10,46^ Furthermore, components in ginger such as alkylated gingerols inhibit the growth of periodontopathogenic gram-negative bacteria, including *Porphyromonas gingivalis* and *Prevotella intermedia*.^38^ The above study results may help explain the statistically significant reduction in gingival bleeding and PD among patients in groups 1 and 2. However, the present results showed statistically significant changes in CAL and MBL in both groups. One clarification for this is that the patients had moderate periodontitis and the follow-up period was relatively short (3 weeks). Such a short follow-up may be insufficient to observe gains in clinical attachment and alveolar bone height. It is hypothesised that local delivery of ginger and multiple sessions of postoperative ginger therapy may facilitate clinical attachment gains and reduction in MBL. Further well-designed and power-adjusted RCTs are needed to test this hypothesis.

One limitation of the present study is that it all participants had moderate periodontitis (stage II/Grade B). This could possibly be associated with the mean ages and inclusion criteria. In the present study, participants in both groups were approximately 45 years old. Non-combustible and combustible tobacco-product users, alcohol users and individuals with self-reported systemic diseases such as DM and AIDS/HIV were excluded. It has been reported that periodontal inflammatory parameters are worse in older patients (≥ 60 years old) and patients with an immunosuppressed health status compared with systemically healthy and younger adults (45 to 49 years old).^2,22,23,25^ Moreover, habitual smoking is known to compromise the outcomes of oral therapeutic interventions.^16,24,36^ Such factors may limit the analgesic effectiveness of traditional NSAIDs and herbal medications, e.g. ginger. Furthermore, it is likely that outcomes of NSPT would be compromised and postoperative self-perceived pain worse in the former than in the latter latter patient group. Due to limitations in funding laboratory-based investigations, assessment of subgingival microbes and levels of proinflammatory cytokines in gingival crevicular fluid and/or whole saliva could not be assessed. It is hypothesised ginger supplementation after NSPT would be more effective in reducing the counts of periodontopathogenic bacteria and levels of inflammatory cytokines such as interleukins and tumor necrosis factor alpha, than NSPT alone. However, a consensus on whether surgical or nonsurgical mechanical instrumentation is most effective for the management of oral inflammatory conditions has yet to be reached.^28^ Further randomised controlled trials are needed in this regard.

## Conclusion

Ginger and traditional NSAIDs are effective in reducing postoperative pain and inflammation following NSPT in patients with moderate periodontitis.
